# Changes in the attitudes of professors and students of medicine towards arabicizing medical terms in the faculties of medicine: A study from Jordan

**DOI:** 10.1016/j.heliyon.2022.e12022

**Published:** 2022-12-06

**Authors:** Dalal Al-Zubi, Ahmad El-Sharif, Karem H. Alzoubi

**Affiliations:** a Department of English Language and Literature, Al al-Bayt University, Jordan; b Department of Clinical Pharmacy, Jordan University of Science and Technology, Jordan

**Keywords:** Arabicization, Medical terms, Medicine, Language planning, Awareness, Attitudes, Barriers, Jordan

## Abstract

This study aimed to investigate changes in awareness, attitudes, acceptability, and possible barriers to using Arabicized medical terms in teaching and daily communication in medical colleges in Jordan. Using a cross-sectional online survey, medicine students and professors in Jordanian universities were sampled to survey their awareness, attitudes, and barriers toward Arabicized medical terms. Responses to the surveys were quantified into awareness, attitudes, and barriers scores and compared according to the sociodemographic variables of the study sample. Results of the current study showed positive awareness/acceptability and attitudes among medicine students toward Arabicized medical terms. The most common perceived barriers toward Arabicization among medicine students were that teaching and assessments (exams, quizzes, projects, etc.) are in English and the unavailability of valuable medical references that use Arabic terms. Several demographic variables were associated with acceptability, attitudes, and/or barriers toward Arabicized terms among medicine students, including gender, income, place of living, level of medicine study, having more than one mother tongue, and language proficiency. Medicine university professors showed acceptable awareness and generally positive attitudes toward Arabicized terms. The most frequently cited barriers among medicine professors were in concordance with those pointed out by medicine students, which indicates the validity of these barriers. Gender and English language proficiency were the only factors associated with acceptability, attitudes, and/or barriers toward Arabicized terms among medicine university professors. In conclusion, the current study indicated a generally more suitable environment for utilizing Arabicized medical terms, especially when delineating major barriers facing medicine students and professors.

## 1 Introduction

In recent decades, the use of English medical terms instead of Arabic medical terms has been recognized in many Arab-speaking countries in the Middle East, such as Jordan, Egypt, and Saudi Arabia, especially among medical faculties and academic settings (Al Bar and Assuhaimi, 1996; Alshareef et al., 2018; Mizher and Al-Al-abed Al-Haq, 2014; Sabbour et al., 2012). Still, in other countries, e.g., Syria, medicine is effectively and thoroughly taught in Arabic (Al-Kateb, 1999; Tekian and Boulet, 2015). Meanwhile, research shows that there have been substantial efforts among academic institutions in the Arab world towards using the Arabic language in teaching natural and human sciences at the university level, especially medicine. That has been fundamentally materialized through the ‘Arabicization’ of technical, medical terms.

The term ‘Arabicization’ has been defined in many ways. AL-Jawhari (1980) says (“*ta'arrab*,” Arabicize” means “to be similar to Arabs,“; and “he was Arabized after emigration “means” he became an Arab.” His tongue was Arabized, “means “he became an Arab. “Arabizing a foreign name” means that Arabs utter it according to their methods. Accordingly, Arabicization is derived from the word “Arabic,” which is the language spoken by the Arabs. The verb derived from the term Arabicization is ‘to Arabicize,’ which means to transfer into Arabic. Thus, the term refers to Arab people and their culture (Al-Abed Al-Haq, 1998; Al-abed Al-Haq, 2007). Arabicization is also defined as “adopting a word that does not exist in the Arabic language and translating it in a way to make it clear and eloquent for the Arabic-speaking communities” (Elnashar and Abdelrahim, 2015; Ghaneim, 1989). Al-Abed Al-Haq (1998) showed the difference in meaning between the two terms “Arabicization” and “Arabization.” The term ‘Arabization’ is derived from the word “Arab”; thus, it refers to Arab culture and people and has nothing to do with language. On the other hand, the term “Arabicization” is derived from the word “Arabic”; therefore, it has to do with lexis and lexical items in Arabic. AL-Abed AL-Haq used “Arabicization” when referring to Arabic language planning. According to AL-Abed AL-Haq, the importance of Arabicization is to survive the Arabic language from death (Al-Abed Al-Haq, 1998).

Attitudes toward the Arabicization of medicine and medical terms have been extensively studied by Arab researchers. Most research has accentuated the existence of a general trend in most Arab countries that favours the Arabicization of medicine. For example, in Egypt, a study showed that more than half of the medicine students sampled did not consider the English Language an obstacle. In that respect, 44.4% of the students surveyed used to translate English medical terms into Arabic terms for added comprehension, whereas 44.5% of the staff considered education in English as an obstacle to their teaching (Sabbour et al., 2012). In Saudi Arabia, a study showed that students had a positive attitude toward Arabicization and preferred to learn in the Arabic language rather than in the English language (Al Bar and Assuhaimi, 1996). A second study in Saudi Arabia accounted for several difficulties and barriers facing the Arabicization of medical terms, especially by inexperienced translators (e.g., the use of non-descriptive or non-expressive, vague Arabic terms for specific foreign medical terms) (Al Faisal, 2017). In addition, it was found that dental and medical students in Saudi Arabia preferred to study their courses in Arabic with the perceived benefits of facilitating learning and improving their understanding of the topics. In that respect, 41% of the students believed that studying English creates a barrier, whereas 9.9% stated that English does not pose a barrier (Khallof et al., 2019). Despite the positive attitudes toward the Arabicization of medical terms among students, it was reported that Saudi decision-makers had an overwhelming preference for the English language over Arabic for medical education (Alshareef et al., 2018). A study among pre-med and first-year medicine students in Qatar reported that the majority of students endorsed the usefulness of glossary for English-Arabic medical terms and reported that they sometimes translate scientific terms into Arabic (Bendriss and Bendriss, 2015). Moreover, 61% of students in the medicine foundation program in Qatar did not ask about the meanings of English medical terms because of shyness (Bendriss and Bendriss, 2015). In two studies, Al-Asal and Smadi compared the study-medium effect on students learning (Al-Asal and Smadi, 2012, 2014). They used medical students at the University of Damascus/Syria versus Jordan University of Science and Technology/Jordan in terms of their use of Arabicized terms. They concluded that lack of knowledge of the language, mainly the foreign language, is one of the primary reasons for lower information acquisition of scientific terms among students. Based on the successes of the Syrian experience in medical education in Arabic at the University of Damascus (*see* Ghazala, 2012, 2013), authors have stressed that Arabic can cope with the new influx of scientific terms where there are many inactive Arabic terms (or expressions) that can be revived and subsequently employed as equivalents to scientific terms in English.

In 2000, a study conducted in Jordan (Halloush, 2000) showed low acceptability of Arabicization among physicians. It reported that Jordanian physicians had major concerns with using Arabicized medical terms and were, in conclusion, neither prepared nor motivated for Arabicized medical terms (Halloush, 2000). Additionally, a study from Egypt showed that medical staff members thought that Arabization would limit graduates’ ability to compete internationally (Sabbour et al., 2012). A recent study from Saudi Arabia reported a positive attitude of decision-makers toward using English for medical instruction. The lack of medical resources was the main obstacle to the use of Arabic. Decision-makers also expressed support for a future Arabic curriculum once obstacles are overcome (Alshareef et al., 2018). The present study investigated the status of the Arabicization of medical terms among university professors and students in Jordan may have changed in terms of awareness, acceptability, attitudes, and barriers. This study initially hypothesized that a change in the attitudes towards Arabic in Jordan must be perceptible and that there is a positive change in the awareness, attitudes, and level of acceptability towards the Arabicization of medical terms in Jordan. That change is influenced by social factors related to gender, profession, and level of education. Accordingly, this study aimed to investigate awareness, attitudes, and perceived barriers among medicine students and their professors toward Arabicized medical terms and Arabicization. In addition, it correlates that awareness, attitudes, and perceived barriers with demographic and language proficiency variables.

## 2 Methods

This study is a quantitative cross-sectional survey of awareness, attitudes, and barriers among medicine students and professors at all universities in Jordan that have a college of medicine, namely The University of Jordan, Jordan University of Science and Technology, Yarmouk University, The Hashemite University, Mutah University, and Applied Sciences University. Data Collection was carried out from October–November of the academic year 2021/2022.

The sample size of the study was calculated using G-Power 3.1., Universitat Kiel, Germany, based on convenience sample method, small effect size, alpha of 0.05, and power of 0.90. The estimated sample size of students was 852, where 855 students' responses were collected. As for professors, medium effect size, alpha of 0.05, and power of 0.90 were assumed. The estimated sample size for the professors was 185, where 202 professors’ responses were collected. Excluded from the study were subjects with Arabic as not their mother language or those who lived in an English-speaking country for more than 16 years.

The study questionnaire (one version for students and another for professors, see appendices I and II) was systematically developed to reflect subjects’ awareness, attitudes, and berries toward Arabicization among medicine students and professors. The study questionnaire was divided into four sections. The first section included demographics and language proficiency variables of the study subjects, including age, gender, income, place of living, level of education, years of study for students, years of experience for professors, mother language, study language, and Arabic and English proficiency. In the second section of the questionnaire, the awareness of subjects about Arabicization and Arabicized medical terms was assessed by presenting a sample of Arabicized terms to study subjects and examining their awareness as per criteria described by Cooper (1989: 61–62). In the third section of the study questionnaire, attitudes toward Arabicized medical terms were assessed as per items previously used and validated by Al-Abed Al-Haq Al-Essa (Al-abed Al-Haq and Al-Essa, 2016). Major perceived barriers were surveyed in the fourth section of the study questionnaire. The above was assessed via a 5-Likert scale (strongly agree, agree, neutral, disagree, strongly disagree).

The list of Arabicized medical terms was chosen from *The Unified Medical Dictionary* (Fourth edition) (Al-Khayat, 2006), a major medical dictionary approved by the Academy of Arabic language of Jordan. It provides Arabicized medical terms and their English counterparts. The chosen terms were based on a model suggested by (Sager, 1990). Five lists of terms (10 terms/each) were determined using a similar approach for deriving the Arabicized terms. According to this approach, five major themes were identified/utilized when creating language terms needed in the medical field (Sager, 1990). These are ‘anatomical location/description,’ ‘physiological functions,’ ‘disease/pathology-based nomenclature,’ ‘examination tests,’ and ‘surgical procedures and operations (Sager, 1990).

Each theme listed above was represented by ten words, as previously described in (Al-abed Al-Haq and Al-Essa, 2016). The medical terms choice was based on the consensus judgmental opinion of five linguistics experts and five medical sciences experts. The whole study questionnaire and the medical terms list were face validated via a review from English linguistics and medical and biomedical sciences experts. Besides, experts ensured that the research questionnaire questions did not inflict personal or psychological harm on the participant. Hence, the questionnaire was approved by the IRB committee in the Deanship of Postgraduate Studies at Al-Albayt University.

After that, the study questionnaire was piloted on 25 study subjects to ensure the comprehensibility of all items and content validity. Piloted subjects were encouraged to provide feedback about the questionnaire items and their clarity. The study questionnaire was finally adjusted based on the results/comments of the pilot study. Subjects from the pilot study were not included in the final analysis of the study. The final study questionnaire was built into google forms and distributed electronically using social media networks such as WhatsApp, Facebook, etc. and via medical students’ social electronic groups. Professors were approached in person by the authors via phone or e-mail to be asked to fill out the study questionnaire. Electronic informed consent was obtained from all study participants via a compulsory question that required reading the study participation terms and information package before the participant was granted access to the study questionnaire.

Awareness, attitude, and barriers scores were calculated by marking the 5-Likert scale items from 1 through 5 as their relevance to contribute toward higher awareness, more favorable attitude, and being a barrier (5 being the highest) towards Arabicized medical terms. Data analysis was conducted using the Statistical Package for Social Sciences (SPSS version 23). Data were tabulated into frequency tables (*see* ∗∗). Awareness, attitude, and barriers scores were in the form of continuous numerical variables. They also met all the assumptions of the ANOVA test, including data normality as per the Kolmogorov-Smirnov test. Therefore, the indicated variables were compared using an unpaired-student t-test for two groups and one-way ANOVA followed by Tukey's post-test for three groups. Statistical significance was considered at P < 0.05.

## 3 Findings

In this section, we show the results obtained from the questionnaires, including awareness, attitudes, and barriers among medicine students and their professors toward Arabicization. The section is organized into two sub-sections: the first section pertains to awareness, attitudes, and barriers among medicine students, whereas the second section pertains to awareness, attitudes, and barriers among university professors.

### 3.1 Awareness, attitudes, and barriers of medicine students towards Arabicization

Medicine Students (n = 855) were included in the study with a response rate of 99%. The average age of student participants was 22.42 ± 2.71 (mean ± SD) years, and the male: female ratio was 1.03. They were comparably divided between pre-clinical and clinical study years, with about 11% at higher specialty levels. The majority of the students were living in cities and had Arabic as their mother tongue. The majority perceived their Arabic proficiency as excellent, whereas English language proficiency was either excellent or very good among the majority of the students sampled. Details of the demographic and language proficiency characteristics of the sampled medicine students are shown in Table 1.

**Table 1:** Demographic, and language proficiency information among students.

Variable	N	%
**Institution**		
The University of Jordan	154	18.0
The Hashemite University	126	14.7
Jordan University of Science and Technology	240	28.1
Yarmouk University	136	15.9
Mutah University	132	15.4
Applied Sciences University	59	5.90
**Level of study**		
Pre-clinical years (years 1–3)	386	45.1
Clinical years (years 4–7)	360	42.1
Higher Specialty	101	11.8
**Gender**		
Female	416	48.7
Male	431	50.4
**Highest education of parent**		
Less than bachelor	190	22.2
Bachelor	441	51.6
Graduate	216	25.3
**Family income**		
<700 JDs	364	42.6
700-1100 JDs	149	17.4
>1100 JDs	334	39.1
**Place of Living**		
Urban	570	66.7
Suburban	277	32.4
**Mother language**		
Arabic	758	88.7
Another language beside Arabic	89	10.4
**Study language at school**		
Arabic	371	43.4
English	52	6.1
English with Arabic subjects	420	49.1
Other languages	4	0.5
**Arabic language proficiency**		
Excellent	559	65.4
Very good	248	29.0
Good	40	4.7
**English language proficiency**		
Excellent	296	34.6
Very good	372	43.5
Good	179	20.9

**Figure 1:**
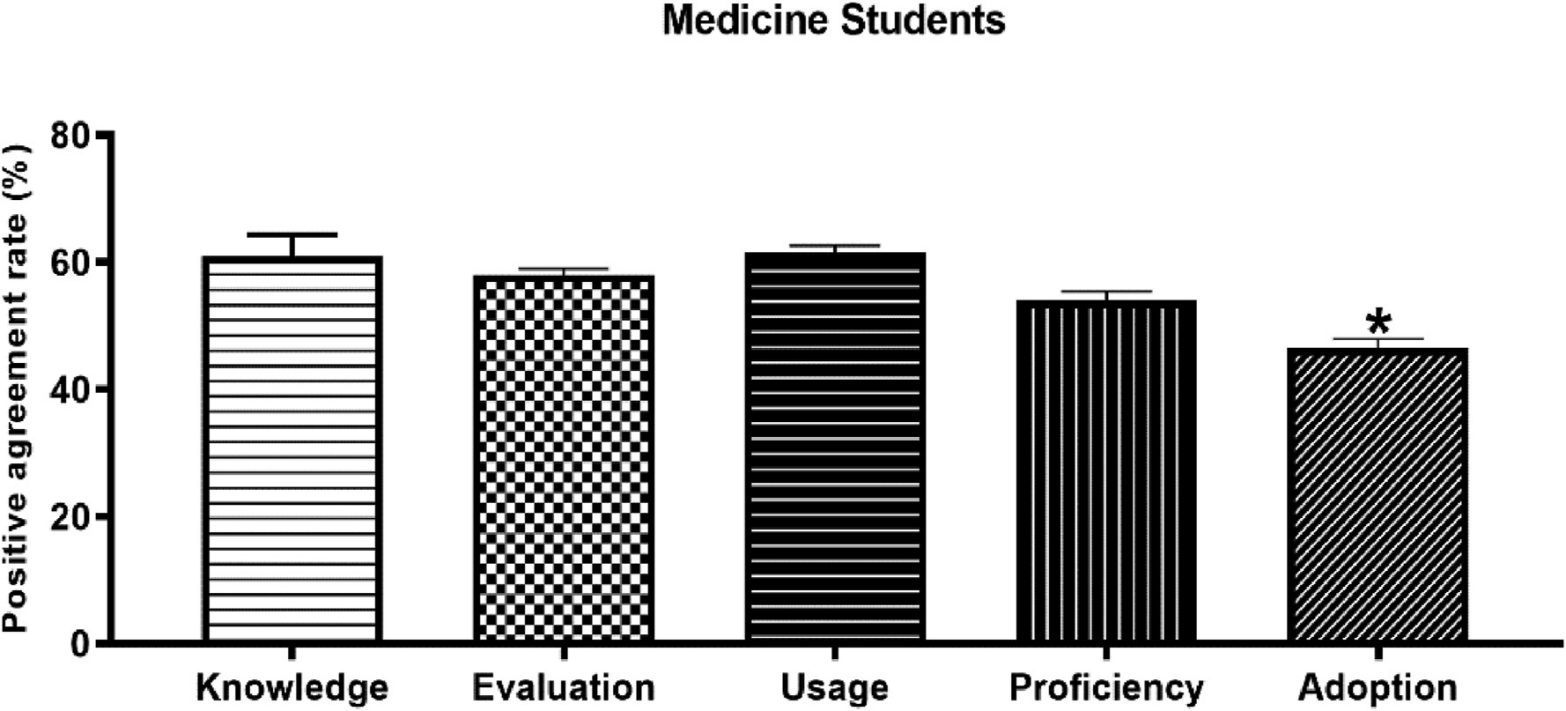
Acceptability of Arabicized medical terms among medicine students as per criteria of Cooper et al., 1989. The overall rate of positive agreement with the terms was generally more than 50%, where medicine students showed significantly lower rates of adoption of Arabicized medical terms compared to knowledge, evaluation, usage, and proficiency. ∗ indicates a significant difference from other groups at p < 0.05.

Table 2 shows the awareness of Arabicized terms per Cooper's criteria (Cooper, 1989) among the sampled students. In general, approximately half of the sample shows positive agreement about their awareness of the presented Arabicized medical terms (Range of percentages: 42.2–67.0%). In that respect, percentages of the positive agreement to knowledge, evaluation, usage, proficiency, and adoption were in the range of 48.1–67.0%, 54.7–60.9%, 58.2–64.9%, 50.3–57.0%, and 42.2–49.1, respectively, as it is illustrated in Figure 1 below.

**Table 2:** Awareness of Arabicized terms as per Cooper's criteria…among students.

Variable	Agree	Strongly Agree	Neutral	Disagree	Strongly disagree
	N(%)	N(%)	N(%)	N(%)	N(%)
***Arabicized terms according to Anatomical location***					
*Knowledge*	*249(19.1)*	248(29.0)	111(13.0)	185(21.0)	54(6.3)
Evaluation	273(31.9)	211(24.7)	147(17.2)	156(18.2)	60(7.0)
Usage	292(34.2)	205(24.0)	112(13.1)	178(20.8)	60(7.0)
Proficiency	243(28.4)	195(22.8)	143(16.7)	171(20.0)	95(11.1)
Adoption	195(22.8)	166(19.4)	119(13.9)	166(19.4)	201(23.5)
***Arabicized terms according to Physiological functions***					
*Knowledge*	289(33.8)	273(31.9)	96(11.2)	136(15.9)	53(6.2)
Evaluation	266(31.1)	230(26.9)	151(17.7)	134(15.7)	66(7.7)
Usage	332(38.8)	223(26.1)	118(13.8)	129(15.1)	45(5.3)
Proficiency	277(32.4)	201(23.5)	147(17.2)	139(16.3)	83(9.7)
Adoption	226(26.4)	184(21.5)	141(16.5)	165(19.3)	131(15.3)
***Arabicized terms according to Disease/pathology-based nomenclature***					
*Knowledge*	292(34.2)	221(25.8)	127(14.9)	149(17.4)	58(6.8)
Evaluation	273(31.9)	195(22.8)	177(20.7)	144(16.8)	58(6.8)
Usage	322(37.7)	183(21.4)	130(15.2)	151(17.7)	61(7.1)
Proficiency	238(27.8)	192(22.5)	182(21.3)	151(17.7)	84(9.8)
Adoption	211(24.7)	165(19.3)	163(19.1)	174(20.4)	134(15.7)
***Arabicized terms according to Examination tests***					
*Knowledge*	338(39.5)	235(27.5)	143(15.7)	102(11.9)	38(4.4)
Evaluation	281(32.9)	239(28.0)	182(21.3)	100(11.7)	45(5.3)
Usage	361(42.2)	180(21.1)	157(18.4)	105(12.3)	44(5.1)
Proficiency	295(34.5)	195(22.8)	161(18.8)	132(15.4)	64(7.5)
Adoption	252(29.5)	170(19.9)	170(19.9)	152(17.8)	103(12.0)
***Arabicized terms according to Surgical procedures and operations***					
*Knowledge*	301(35.2)	244(28.5)	132(15.4)	133(15.6)	37(4.3)
Evaluation	286(33.5)	222(26.0)	176(20.6)	117(13.7)	46(5.4)
Usage	343(40.1)	183(21.4)	152(17.8)	131(15.3)	38(4.4)
Proficiency	275(32.2)	200(23.4)	167(19.5)	143(16.7)	62(7.3)
Adoption	237(27.7)	183(21.4)	164(19.2)	160(18.7)	103(12.0)

The overall rate of positive agreement for adopting Arabicized medical terms among students showed significantly lower rates compared to knowledge, evaluation, usage, and proficiency (*see* Figure 1). No remarkable variations were observed among students’ responses to the subgroups of Arabicized medical terms presented according to Sager (1990).

**Figure 2:**
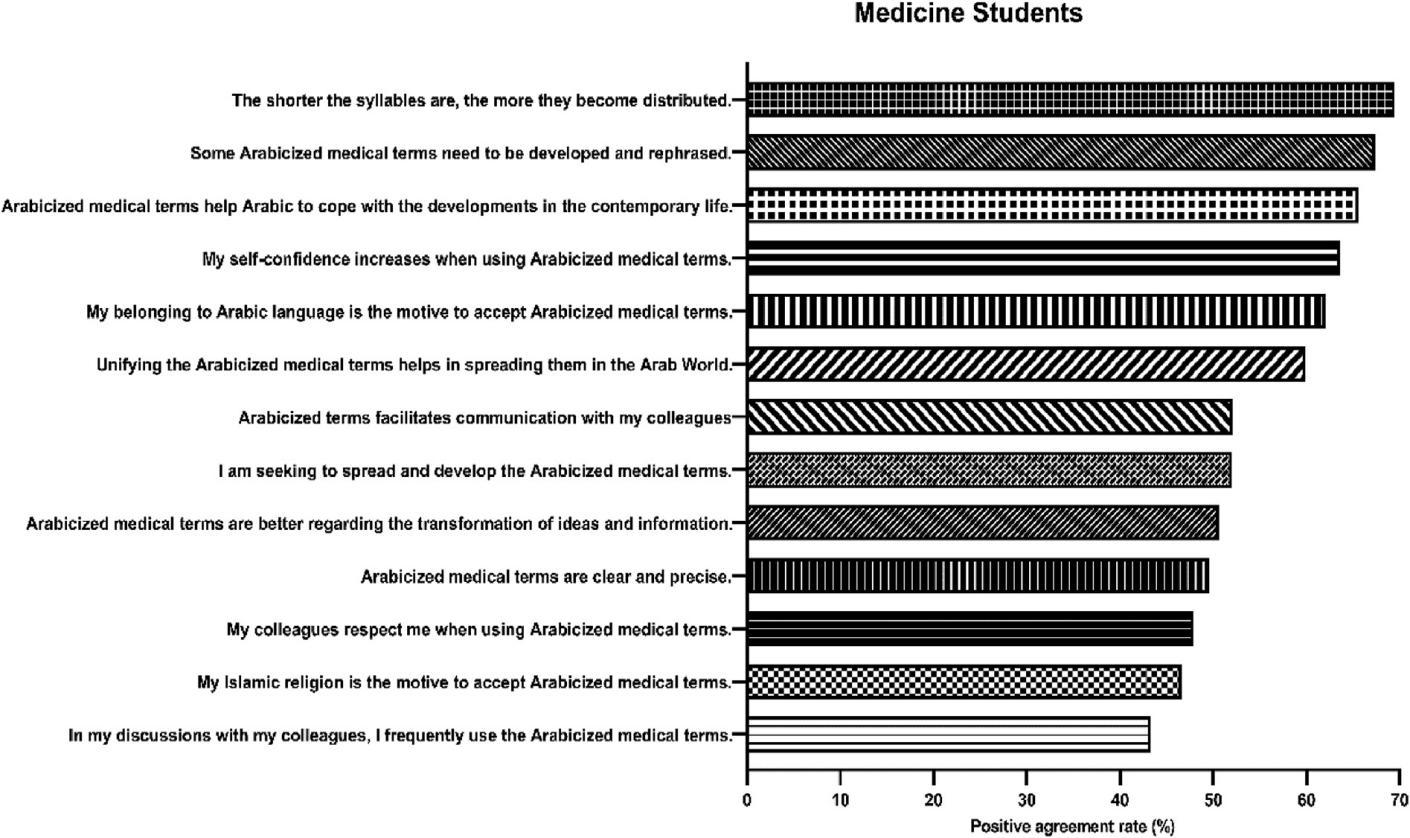
Attitudes of medicine students toward Arabicized medical terms. The most positive attitudes were for the need for shorter syllabi, the need to develop and rephrase some of the Arabicized terms, and that Arabicized terms will help Arabic cope with the developments of contemporary life.

Attitudes of study participants toward Arabicization are shown in Table 3. The majority of students (63.6%) agreed or strongly agreed that their self-confidence increases when using Arabicized medical terms. More than half (59.9%) agreed or strongly agreed with the need to unify the Arabicized medical terms to help them spread all over the Arab World. They also thought that the shorter the syllabi are for the Arabicized medical terms, the more they become distributed (69.8%). Some Arabicized medical terms need to be developed and rephrased (67.4%). Finally, 65.6% of the students thought that the Arabicized medical terms help the Arabic language to cope with the developments in contemporary life (*see* Figure 2 below).

**Table 3:** Attitudes toward Arabicized terms among students.

Statement	Agree	Strongly Agree	Neutral	Disagree	Strongly disagree
	N(%)	N(%)	N(%)	N(%)	N(%)
My self-confidence increases when using Arabicized medical terms.	*185(21.6)*	359(42.0)	129(15.1)	115(13.5)	59(6.9)
My belonging to the Arabic language is the motive to accept Arabicized medical terms.	278(32.5)	252(29.5)	155(18.1)	111(13.0)	51(6.0)
My Islamic religion is the motive to accept Arabicized medical terms.	192(22.5)	206(24.1)	216(25.3)	144(16.8)	89(10.4)
I think that The Arabicized business terms are better than the English business terms regarding the transformation of ideas and information. I think that Arabicized medical terms are better than English medical terms regarding the transformation of ideas and information.	191(22.3)	242(28.3)	160(18.7)	134(15.7)	120(14.0)
I think that Arabicized terms facilitate communication with my colleagues	234(27.4)	211(24.7)	140(16.4)	141(16.5)	121(14.2)
I think that unifying the Arabicized medical terms helps in spreading them in the Arab World.	283(33.1)	229(26.8)	195(22.8)	80(9.4)	60(7.0)
I think that the shorter the syllables are for the Arabicized medical terms the more they become distributed.	310(36.3)	284(33.2)	146(17.1)	62(7.3)	45(5.3)
I think that the Arabicized medical terms are clear and precise.	229(26.8)	195(22.8)	211(24.7)	129(15.1)	83(9.7)
I think that some of the Arabicized medical terms need to be developed and rephrased.	301(35.2)	275(32.2)	191(22.3)	40(4.7)	4.(4.7)
My colleagues respect me when using Arabicized medical terms.	213(24.9)	197(23.0)	273(31.9)	102(11.9)	62(7.3)
I think that Arabicized medical terms help the Arabic language cope with the developments in contemporary life.	329(38.5)	232(27.1)	170(19.9)	67(8.0)	48(5.6)
In my discussions with my colleagues, I frequently use Arabicized medical terms.	199(23.3)	171(20.0)	190(22.2)	144(16.8)	143(16.7)
I am seeking to spread and develop Arabicized medical terms.	240(28.1)	204(23.9)	217(25.4)	93(10.9)	93(10.9)

**Figure 3:**
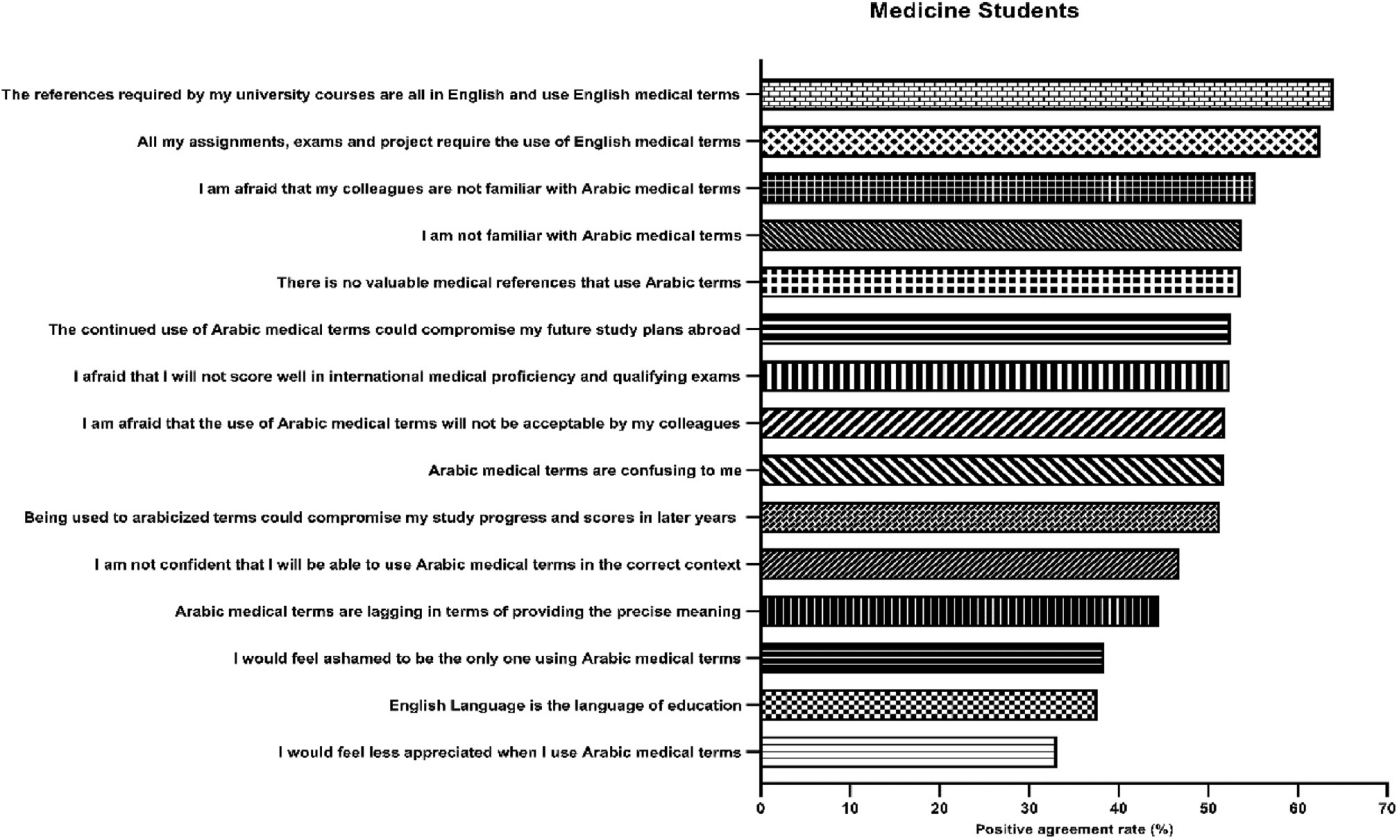
Barriers of medicine students toward Arabicized medical terms. The most common two barriers were that the references of university courses are in English and that all the assignments and projects in university courses require English medical terms.

As for barriers related to the use of Arabicized medical terms among medicine students, the major barriers that were agreed or strongly agreed on were that current assignments, exams, and projects require the use of English medical terms (62.5%), and the unavailability of valuable medical references that use Arabic terms (62.0%) where all references required by university courses in medical colleges are all in English and use English medical terms (64.0%) as it is shown in Table 4. Other barriers where the positive agreement was about 50% of the students included unfamiliarity of the student himself (53.7%) or perception of the unfamiliarity of his colleagues (53.9%) with the Arabicized medical terms, the terms being confusing (51.9%), being worried that the terms will not be acceptable by colleagues (51.9%), concern that the continued use of Arabicized medical terms could compromise student's study progress (51.3%), scores in international exams (52.4%), and future study plans abroad (52.5%), as Table (7), and Figure 3, below illustrate.

**Table 4:** Barrier toward Arabicization medical terms among students.

Statement	Agree	Strongly Agree	Neutral	Disagree	Strongly disagree
	N(%)	N(%)	N(%)	N(%)	N(%)
I am not familiar with Arabic medical terms	226(26.4)	233(27.3)	122(14.3)	162(18.9)	104(12.2)
I am afraid that my colleagues are not familiar with Arabic medical terms	248(29.0)	225(26.3)	151(17.7)	177(20.7)	46(5.4)
I would feel less appreciated when I use Arabic medical terms	135(13.8)	165(19.3)	178(20.8)	252(29.5)	117(13.7)
Arabic medical terms are lagging in terms of providing the precise meaning	179(20.9)	202(23.6)	191(22.3)	192(22.5)	83(9.7)
Arabic medical terms are confusing to me	202(23.6)	240(28.1)	169(19.8)	177(20.7)	59(6.9)
I am not confident that I will be able to use Arabic medical terms in the correct context	196(22.9)	204(23.9)	194(22.7)	180(21.1)	73(8.5)
I am afraid that the use of Arabic medical terms will not be acceptable to my colleagues	207(24.2)	237(27.7)	199(23.3)	145(17.0)	59(6.9)
I would feel ashamed to be the only one using Arabic medical terms	164(19.2)	165(19.2)	213(24.9)	224(26.2)	81(9.5)
The English Language is the language of education	151(17.7)	170(19.9)	227(26.5)	203(23.7)	96(11.2)
Being used to Arabic medical terms could compromise my study progress and scores in later years as all my future study is expected to be using English medical terms	201(23.5)	238(27.8)	199(23.3)	153(17.9)	56(6.5)
All my assignments, exams, and project require the use of English medical terms	193(22.6)	341(39.9)	155(18.1)	123(14.4)	35(4.1)
There are no valuable medical references that use Arabic terms	186(21.8)	272(31.8)	214(25.0)	137(16.0)	38(4.4)
The references required by my university courses are all in English and use English medical terms	181(21.2)	366(42.8)	153(17.9)	113(13.2)	34(4.0)
I am afraid that I will not score well in international medical proficiency and qualifying exams	190(22.2)	264(30.2)	204(23.9)	153(17.9)	36(4.2)
The continued use of Arabic medical terms could compromise my future study plans abroad	194(22.7)	255(29.8)	193(22.6)	159(18.6)	46(5.4)

The scores for students’ awareness, attitudes, and barriers were compared per demographic and educational characteristics, as shown in Table (5). For the level of student study, those at higher specialty level had significantly (P < 0.05) higher awareness and lower barrier scores than those in pre-clinical or clinical phases of medicine study. Additionally, higher specialty students had significantly (P < 0.05) more positive attitudes than those in clinical study years. Moreover, those in clinical study years had significantly (P < 0.05) more positive attitudes and lower perceived barriers than students in the pre-clinical years. Male students had significantly higher awareness (P = 0.006) and more positive attitude (P < 0.001) scores compared to female students. As for family income, those who reported higher family income had significantly (P < 0.05) lower barrier scores compared to the middle-income and lower-income categories. Moreover, those in the middle-income category had significantly (P < 0.05) lower barrier scores than those in the lower-income category. Living in the suburbs was associated with significantly (P = 0.044) more positive attitudes toward Arabicized medical terms.

As for the effect of bilingualism (Table 5), having another language besides Arabic as a mother tongue resulted in significantly more awareness (P = 0.002) and more positive attitude (P < 0.001), yet more barriers (P < 0.001) compared to those with only Arabic as a mother tongue. On the other hand, students who had their school years in a mixed English and Arabic medium had significantly better awareness (P < 0.05), yet less positive attitude (P < 0.05), and more barriers (P < 0.05) compared to those with pure Arabic or English school study medium. In terms of Arabic language proficiency, those who reported excellent proficiency in standard Arabic had significantly higher awareness (P < 0.05) and more positive attitudes (P < 0.05) toward Arabicized medical terms as compared to those who reported very good or good Arabic language proficiency. Moreover, those who reported good proficiency in Arabic had significantly lower awareness (P < 0.05) and less positive attitudes (P < 0.05) toward Arabicized medical terms as compared to those who reported very good Arabic language proficiency. As for barriers, excellent and good Arabic language proficient students had lower barrier scores (P < 0.05) versus those with very good proficiency. Finally, students who reported excellent English had better awareness (P < 0.05) and more positive attitudes (P < 0.05) toward Arabicization compared to those with good or very good English proficiency. As for barriers toward Arabicization, the scores were significantly higher (P < 0.05) among students who reported excellent English proficiency.

**Table 5:** Total awareness, attitude, and perceived barriers scores as per demographic and education characteristics of the study sample.

Variable	Awareness		Attitude		Barriers	
	Mean ± SD	P-value	Mean ± SD	P-value	Mean ± SD	P-value
**Level of study**		**<0.001**		**0.005**		**<0.001**
Pre-clinical years (years 1–3)	85.97 ± 26.20		46.87 ± 12.93		56.17 ± 12.65	
Clinical years (years 4–7)	84.30 ± 23.28		44.43 ± 12.53^a^		50.04 ± 14.30^a^	
Higher Specialty	98.77 ± 16.89^a^		48.08 ± 9.58^c^		39.13 ± 11.96^a^	
**Gender**		**0.006**		**<0.001**		0.551
Female	84.43 ± 23.69		43.98 ± 12.14		51.23 ± 13.39	
Male	89.07 ± 24.92		47.90 ± 12.49		51.82 ± 15.20	
**Highest education of the parent**		0.051		0.639		**0.037**
Less than bachelor	46.45 ± 10.38		50.83 ± 13.32		85.27 ± 21.88	
Bachelor	46.69 ± 12.99		51.95 ± 15.06		88.78 ± 24.97	
Graduate	44.10 ± 12.92		51.29 ± 13.76		84.05 ± 25.14^c^	
**Family income**		**<0.001**		**<0.001**		**<0.001**
<700 JDs	48.16 ± 12.58		54.21 ± 14.27		90.37 ± 25.17	
700-1100 JDs	46.11 ± 11.63		48.83 ± 14.72		87.33 ± 22.08^b^	
>1100 JDs	43.53 ± 12.28		49.81 ± 13.78^b^		82.64 ± 24.00^a^	
**Place of Living**		0.926		**0.044**		0.586
Urban	86.84 ± 24.83		45.37 ± 12.87		51.72 ± 14.12	
Suburban	86.68 ± 23.60		47.21 ± 11.52		51.14 ± 14.79	
**Mother language**		**0.002**		**<0.001**		**<0.001**
Arabic	85.30 ± 23.91		45.52 ± 12.25		50.93 ± 14.13	
Another language beside Arabic	99.44 ± 25.14		49.84 ± 13.62		56.67 ± 15.15	
**Study language at school**		**<0.001**		**<0.001**		**<0.001**
Arabic	42.35 ± 12.63		54.16 ± 11.20		80.21 ± 22.13	
English	41.35 ± 13.61		56.77 ± 10.24		73.63 ± 27.31	
English with Arabic subjects	49.67 ± 11.03^a^		48.55 ± 16.48^a^		94.11 ± 23.77^a^	
**Arabic language proficiency**		**<0.001**		**<0.001**		**<0.001**
Excellent	91.38 ± 25.75		47.70 ± 13.44		54.02 ± 14.87	
Very good	79.26 ± 18.11^a^		43.43 ± 9.12^a^		45.35 ± 11.72^a^	
Good/acceptable	69.28 ± 19.76^b^		37.68 ± 10.34^b^		55.03 ± 8.99	
**English language Proficiency**		**<0.001**		**<0.001**		**0.027**
Excellent	97.80 ± 25.33^a^		49.27 ± 13.70^a^		53.03 ± 16.77	
Very good	82.27 ± 21.70		44.12 ± 11.74		49.88 ± 13.39^b^	
Good	77.97 ± 21.60		44.37 ± 10.59		52.48 ± 11.28	

P-values <0.05 indicates significant difference.

a Indicates significant difference from all other groups.

b indicates significant difference from the first group.

c indicates significant difference from the second group.

### 3.2 Awareness, attitudes, and barriers of medicine professors towards Arabicization

The sampled medicine professors were n = 202, with a mean age of 38.12 ± 13.34. Most had work years of experience of more than five years (83.7%), have Arabic as their mother language (92.4%), and English as their study or training language (91.1%). The majority reported excellent Arabic (83.8%) and English (60.4%) language proficiency. Other demographic and language proficiency characteristics of the study sample are shown in Table 6. Awareness of professors (the Cooper criteria (Cooper, 1989),) toward Arabicized terms as categorized by Sager (1990) are shown in Table 7.

**Table 6:** Demographic, and language proficiency information among professors.

Variable	N	%
**Institution**		
The University of Jordan	32	16.2
The Hashemite University	31	15.7
Jordan University of Science and Technology	77	39.1
Yarmouk University	28	14.2
Mutah University	28	14.2
**Years of Experience**		
<5 years	32	16.2
>5–15 years	81	41.1
>15 years	84	42.6
**Gender**		
Female	55	27.9
Male	142	72.1
**Mother Language**		
Arabic	182	92.4
Non-Arabic	15	7.6
**Study/training language**		
English	181	91.9
Non-English	16	8.1
**Language of study/training country**		
English	136	69.0
Arabic	51	25.9
Others	10	5.1
**Arabic language Proficiency**		
Excellent	165	83.8
Very good	27	13.7
Good	5	2.5
**English language Proficiency**		
Excellent	119	60.4
Very good	60	30.5
Good	18	9.1

**Table 7:** Awareness of Arabicized terms as per Cooper's criteria among professors.

Variable	Agree	Strongly Agree	Neutral	Disagree	Strongly disagree
	N(%)	N(%)	N(%)	N(%)	N(%)
***Arabicized terms according to Anatomical location***					
*Knowledge*	76(38.6)	26(13.2)	25(12.7)	56(28.4)	14(7.1)
Evaluation	78(39.6)	14(7.1)	49(24.9)	35(17.8)	21(10.7)
Usage	80(40.6)	17(8.6)	28(14.6)	52(26.6)	20(10.2)
Proficiency	53(26.9)	8(4.1)	30(15.2)	67(38.6)	30(15.2)
Adoption	17(8.6)	5(2.5)	30(15.2)	78(39.6)	67(34.0)
***Arabicized terms according to Physiological functions***					
*Knowledge*	82(41.6)	34(17.3)	24(12.2)	44(22.3)	13(6.6)
Evaluation	63(32.0)	18(9.1)	46(23.4)	50(25.4)	20(10.2)
Usage	83(42.1)	29(14.7)	28(14.2)	47(23.9)	10(5.1)
Proficiency	60(30.5)	17(8.6)	27(13.7)	63(32.0)	30(15.2)
Adoption	34(17.3)	9(4.6)	28(14.2)	80(40.6)	46(23.4)
***Arabicized terms according to Disease/pathology-based nomenclature***					
*Knowledge*	74(37.6)	23(11.7)	31(15.7)	56(28.4)	13(6.6)
Evaluation	66(33.5)	13(6.6)	41(20.8)	57(28.9)	20(10.2)
Usage	73(37.1)	21(10.7)	36(18.3)	52(26.4)	15(7.6)
Proficiency	53(26.9)	15(7.6)	33(16.8)	63(32.0)	33(16.8)
Adoption	31(15.7)	8(4.1)	28(14.2)	76(38.6)	54(27.4)
***Arabicized terms according to Examination tests***					
*Knowledge*	104(52.8)	33(16.8)	22(11.2)	30(15.2)	8(4.1)
Evaluation	75(38.1)	30(15.2)	44(22.3)	34(17.3)	14(7.1)
Usage	105(53.3)	31(15.7)	24(12.2)	29(14.7)	8(4.1)
Proficiency	71(36.0)	27(13.7)	29(14.7)	42(21.3)	28(14.2)
Adoption	53(26.9)	15(7.5)	25(12.7)	65(33.0)	39(19.8)
***Arabicized terms according to Surgical procedures and operations***					
*Knowledge*	90(45.7)	33(16.8)	33(16.8)	31(15.7)	10(5.1)
Evaluation	73(37.1)	25(12.7)	47(23.9)	35(17.8)	17(8.6)
Usage	86(43.7)	32(16.2)	29(14.7)	38(19.3)	12(6.1)
Proficiency	71(36.0)	24(12.2)	30(15.2)	48(24.4)	24(12.2)
Adoption	46(23.4)	17(8.6)	28(14.2)	65(33.0)	41(20.8)

**Figure 4:**
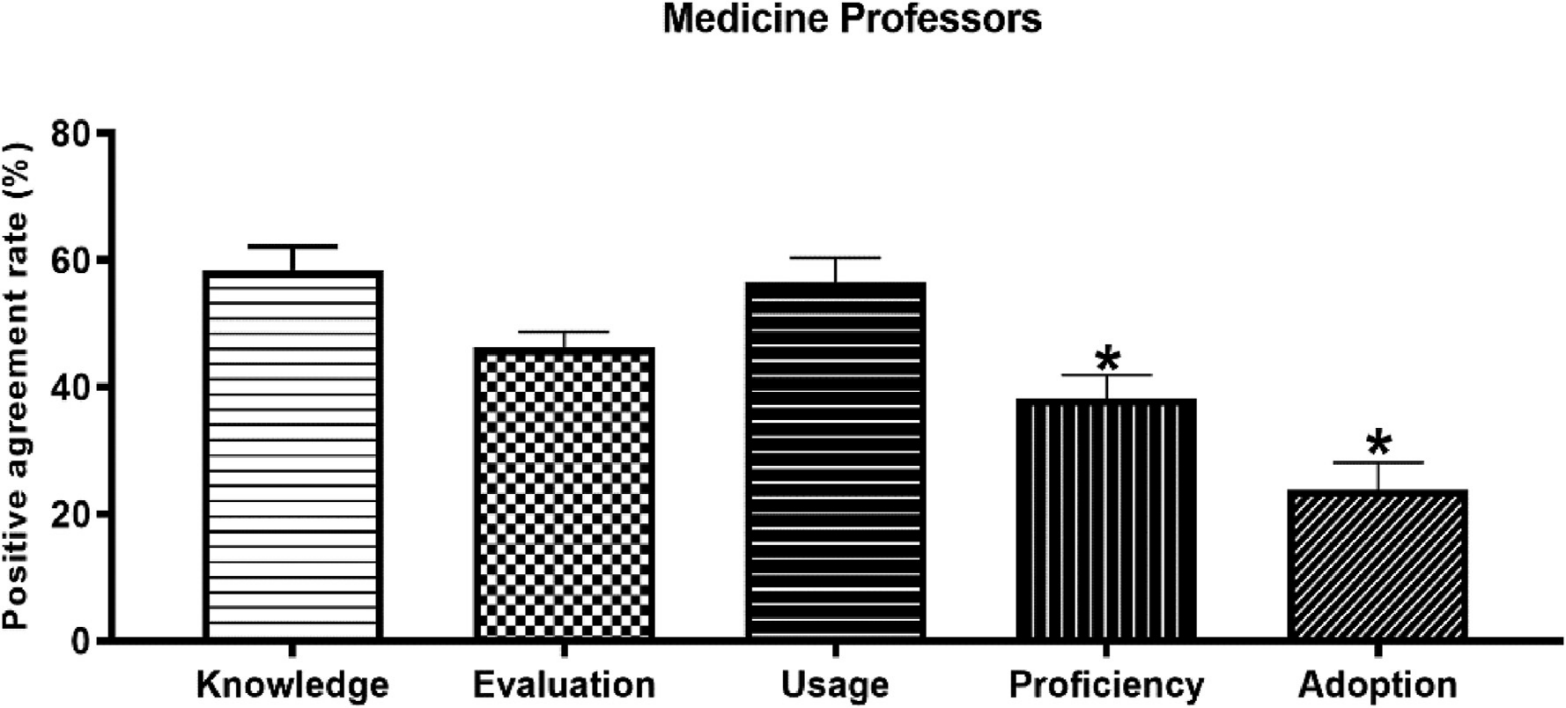
Acceptability of Arabicized medical terms among medicine Professors as per criteria of Cooper et al., 1989. The overall rate of positive agreement with the terms was generally more than 50%. Medicine professors showed significantly lower rates of adoption and proficiency of Arabicized medical terms compared to knowledge, evaluation, and usage. ∗ indicates a significant difference from other groups at p < 0.05.

Agreement or strong agreement with knowledge was highest for terms Arabicized as per medical examination tests (69.1%) and lowest for terms Arabicized as per disease/pathology-based nomenclature (49.3%). In general, significantly lower rates of positive agreement for Arabicized medical terms were reported for adoption and proficiency compared to knowledge, evaluation, and usage criteria among medicine university professors, as illustrated in Figure (4) below.

**Figure 5:**
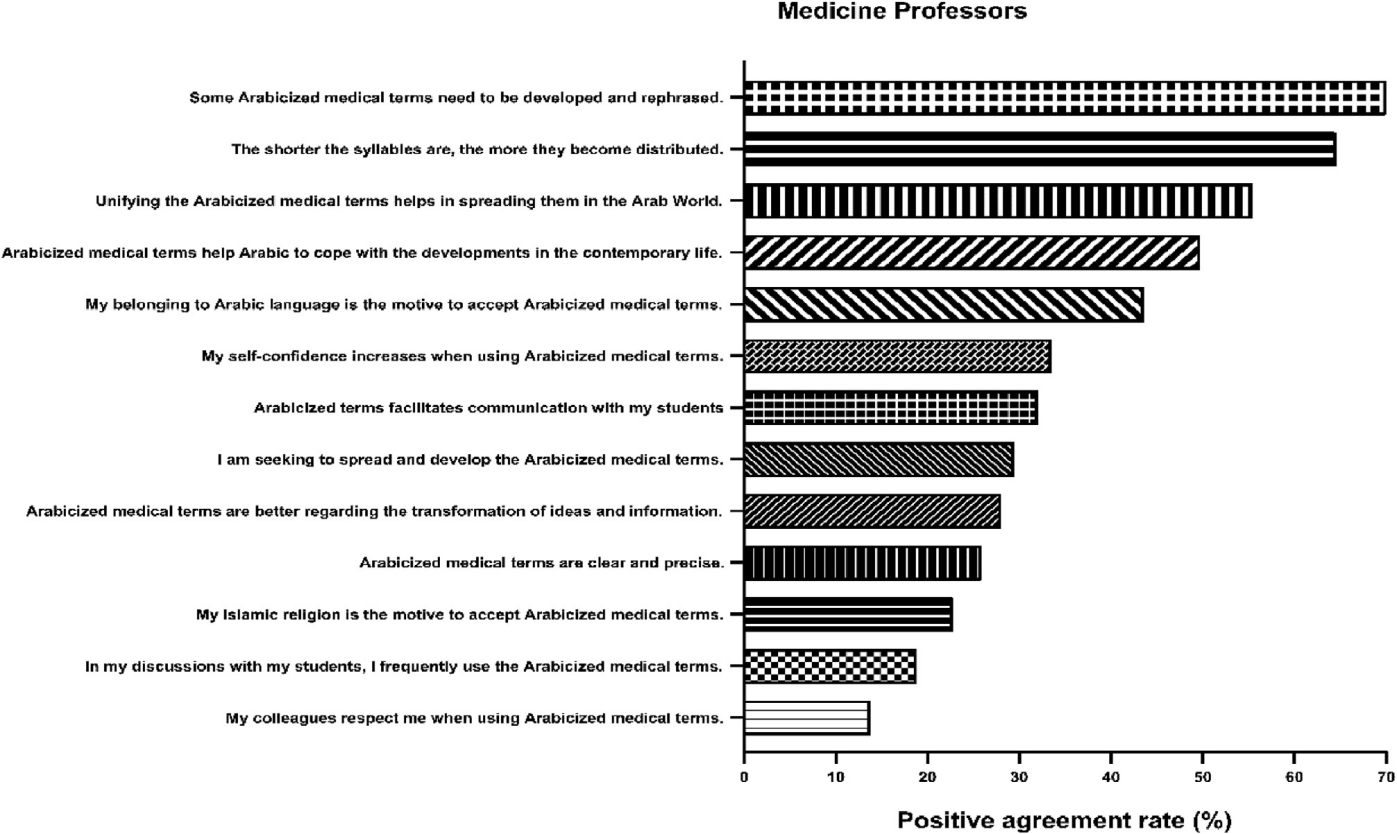
Attitudes of medicine professors toward Arabicized medical terms. The most positive attitudes were for the need to develop and rephrase some of the Arabicized terms, the need to use shorter syllabi in Arabicized medical terms, and the necessity to unify Arabicized medical terms to help them spread all over the Arab world.

As for attitudes toward Arabicized medical terms, the majority of professors agreed or strongly agreed that adaptation of shorter syllabi aids the spread of Arabicized medical terms (64.3%) and that more terms need to be developed and rephrased (70.0%). Other attitude items are shown in Table 8 and illustrated in Figure 5 below.

**Table 8:** Attitudes toward Arabicized terms among professors.

Statement	Agree	Strongly Agree	Neutral	Disagree	Strongly disagree
	N(%)	N(%)	N(%)	N(%)	N(%)
My self-confidence increases when using Arabicized medical terms.	21(10.7)	45(22.8)	37(18.8)	74(37.6)	20(10.2)
My belonging to the Arabic language is the motive to accept Arabicized medical terms.	56(28.4)	30(15.2)	43(21.8)	49(24.9)	19(9.6)
My Islamic religion is the motive to accept Arabicized medical terms.	26(13.2)	19(9.6)	58(29.4)	66(33.5)	28(14.2)
I think that Arabicized medical terms are better than English medical terms regarding the transformation of ideas and information.	35(17.8)	20(10.2)	34(17.3)	73(37.1)	35(17.8)
I think that Arabicized terms facilitate communication with my students	50(25.4)	13(6.6)	36(18.3)	63(32.0)	35(17.8)
I think that unifying the Arabicized medical terms helps in spreading them in the Arab World.	63(32.0)	46(23.4)	32(16.2)	36(18.3)	20(10.2)
I think that the shorter the syllables are for the Arabicized medical terms the more they become distributed.	90(45.7)	37(18.8)	33(16.8)	22(11.2)	15(7.6)
I think that the Arabicized medical terms are clear and precise.	37(18.8)	14(7.1)	49(24.9)	69(35.0)	28(14.2)
I think that some of the Arabicized medical terms need to be developed and rephrased.	82(41.6)	56(28.4)	29(14.7)	20(10.2)	10(5.1)
My colleagues respect me when using Arabicized medical terms.	20(10.2)	7(3.6)	83(42.1)	66(33.5)	21(10.7)
I think that Arabicized medical terms help the Arabic language cope with the developments in contemporary life.	70(35.5)	28(14.2)	46(23.4)	38(19.3)	15(7.6)
In my discussions with my students, I frequently use Arabicized medical terms.	29(14.7)	8(4.1)	43(21.8)	85(43.1)	32(16.2)
I am seeking to spread and develop Arabicized medical terms.	44(22.3)	14(7.1)	63(32.0)	43(21.8)	33(16.8)

**Figure 6:**
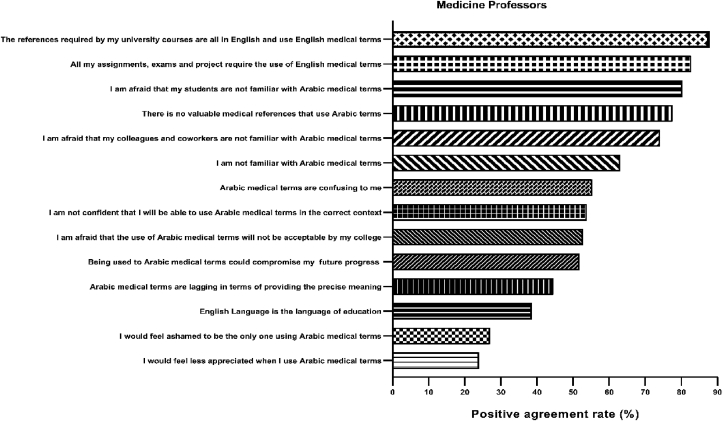
Barriers of medicine professors toward Arabicized medical terms. The most common two barriers were that the references of university courses are in English and that all the assignments and projects in university courses/work requires English medical terms.

Barriers to the Arabicization of medical terms among medicine professors are shown in Table 9. The most prominent barriers toward Arabicized terms, offering a high rate of agreement or strong agreement among professors, were that the references used in university courses are all in English and use English medical terms (87.8%), the fact that all assignments, exams, and project require the use of English medical terms (82.8%), concern that students are not familiar with Arabic medical terms (80.2%), and unavailability of medical references that use Arabic terms (77.6%). Items that received the lowest agreement were feeling less appreciated (23.9%) or ashamed (27.0%) when using Arabic medical terms (*see* Figure (6) below).

**Table 9:** Barrier toward Arabicization medical terms among professors.

Statement	Agree	Strongly Agree	Neutral	Disagree	Strongly disagree
	N(%)	N(%)	N(%)	N(%)	N(%)
I am not familiar with Arabic medical terms	89(45.2)	35(17.8)	30(15.2)	37(18.8)	6(3.0)
I am afraid that my colleagues and coworkers are not familiar with Arabic medical terms	107(54.3)	39(19.8)	25(12.7)	24(12.2)	2(1.0)
I am afraid that my students are not familiar with Arabic medical terms	112(56.9)	46(23.4)	21(10.7)	14(7.1)	4(2.0)
I would feel less appreciated when I use Arabic medical terms	35(17.8)	12(6.1)	65(33.0)	66(33.5)	19(9.6)
Arabic medical terms are lagging in terms of providing the precise meaning	57(28.9)	31(15.7)	49(24.9)	44(22.3)	16(8.1)
Arabic medical terms are confusing to me	72(36.5)	37(18.8)	41(20.8)	35(17.8)	12(6.1)
I am not confident that I will be able to use Arabic medical terms in the correct context	72(36.5)	34(17.3)	42(21.3)	37(18.8)	12(6.1)
I am afraid that the use of Arabic medical terms will not be acceptable to my college	75(38.1)	29(14.7)	54(27.4)	34(17.3)	5(2.5)
I would feel ashamed to be the only one using Arabic medical terms	33(16.8)	20(10.2)	50(25.4)	75(38.1)	19(9.6)
The English Language is the language of education	54(27.4)	22(11.2)	41(20.8)	62(31.5)	18(9.1)
Being used to Arabic medical terms could compromise my study progress and scores in later years as all my future study is expected to be using English medical terms	63(32.0)	39(19.8)	37(18.8)	43(21.8)	15(7.6)
All my assignments, exams, and project require the use of English medical terms	86(43.7)	77(39.1)	19(9.6)	12(6.1)	3(1.5)
There are no valuable medical references that use Arabic terms	58(29.4)	95(48.2)	24(12.2)	12(6.1)	8(4.1)
The references required by my university courses are all in English and use English medical terms	56(28.4)	117(59.4)	10(5.1)	8(4.1)	6(3.0)

The awareness, attitude, and perceived barriers scores were compared as per demographic and education characteristics of the professors’ sample (Table 10). Gender was significant, where female professors had higher awareness (P < 0.001) and more positive attitude (P < 0.001) scores compared to their male counterparts. As for English language proficiency, those who perceived their English proficiency as “Excellent” had significantly (P < 0.05) more positive attitudes toward Arabicized medical terms than those who perceived it as “Good.”

**Table 10:** Total awareness, attitude, and perceived barriers scores as per demographic and education characteristics of the study sample.

Variable	Awareness		Attitude		Barriers	
	Mean ± SD	P-value	Mean ± SD	P-value	Mean ± SD	P-value
**Years of Experience**		0.978		0.628		0.078
<5 years	73.2 ± 20.88		38.88 ± 12.72		31.44 ± 9.75	
>5–15 years	74.15 ± 21.61		38.47 ± 11.52		36.06 ± 10.36	
>15 years	73.77 ± 21.08		40.19 ± 11.52		34.58 ± 9.15	
**Gender**		**<0.001**		**<0.001**		0.636
Male	70.54 ± 20.99		37.39 ± 11.59		34.89 ± 9.51	
Female	82.35 ± 19.30		44.11 ± 10.60		34.15 ± 10.71	
**Mother Language**		0.302		0.325		0.483
Arabic	73.40 ± 21.20		39.03 ± 11.70		34.54 ± 9.51	
Non-Arabic	79.27 ± 20.679		42.13 ± 11.58		36.40 ± 13.50	
**Study/training language**		0.67		0.924		0.856
English	74.66 ± 21.19		39.29 ± 11.87		34.72 ± 9.86	
Non-English	64.56 ± 19.03		39.00 ± 9.75		34.25 ± 9.88	
**Language of study/training country**		0.551		0.206		0.171
English	73.70 ± 19.62		38.32 ± 10.67		35.47 ± 9.96	
Arabic	75.45 ± 24.90		41.73 ± 13.98		32.45 ± 9.62	
Others	67.50 ± 21.81		39.70 ± 11.61		35.30 ± 8.19	
**Arabic Language Proficiency**		0.944		0.101		0.412
Excellent	73.66 ± 21.22		39.67 ± 12.00		35.07 ± 10.20	
Very good	74.41 ± 19.60		35.56 ± 8.64		32.96 ± 7.12	
Good	76.60 ± 31.18		46.00 ± 12.17		31.00 ± 10.27	
**English Language Proficiency**		0.186		**0.006**		0.765
Excellent	75.85 ± 20.52		41.41 ± 11.81		35.08 ± 10.48	
Very good	71.83 ± 22.32		36.23 ± 11.44		33.95 ± 8.92	
Good	67.22 ± 20.55		35.22 ± 8.66#		34.44 ± 8.41	

# P-values <0.05 indicates significant difference from other groups.

## 4 Discussion

The current study is a synchronic study investigating changes in awareness, attitude, and barriers toward Arabicized medical terms among medicine students and their professors. The current study showed that medicine students’ awareness of Arabicized medical terms ranged from 42.2% to 67.0%. Thus, an improvement in awareness and more positive attitudes toward Arabicized medical terms were shown. Several barriers to Arabization were reported.

### 4.1 Awareness of Arabicization

As for medicine university professors, they showed the highest knowledge of Arabicized terms as per examination tests (69.1%) and the lowest for Arabicized terms as per disease/pathology-based nomenclature (49.3%). This could be related to the fact that terms related to medical examination/diagnostic tests must be frequently referred to in Arabic or as Arabicized terms when dealing with patients as opposed to those related to disease pathology. Thus, they may have more restricted use among medical professionals. As expected, lower awareness was reported for other items in the Cooper acceptability scale (Cooper, 1989): evaluation, usage, and adoption, with the lowest scores for adoption (11.1–34.4%). This is true as adoption represents the most advanced stage of utilizing Arabicized medical terms (Cooper, 1989).

### 4.2 Attitudes toward Arabicization

In the current study, medicine students indicated the need to unify Arabicized medical terms to ensure their spread all over the Arab world. They also noted the need to revise and rephrase many Arabicized medical terms. They indicated that the shorter the syllabi are for the Arabicized medical terms, the more they become distributed. This is in concordance with the results of medicine professors who reported the majority that adaptation of shorter syllabi aids the spread of Arabicized medical terms and that more Arabicized terms need to be developed and rephrased. Results by Sabbour et al. (2012) concordance between medicine students and staff Arabization will result in a strange language with difficult Arabic terminology which will be different from patients’ everyday language. Moreover, current results align with those of business students who had the highest attitude scores for the need to develop and rephrase several of the Arabicized business terms and the need to use shorter syllabi and phrases (Al-Al-Abed Al-Haq and Al-Essa, 2016).

### 4.3 Barriers to Arabicization

The major perceived barriers toward Arabicized medical terms among medicine students included teaching and assessments (exams, quizzes, projects, etc.) in English and the unavailability of valuable medical references that use Arabic terms. This correlates with the views pointed out by medical students and their teaching staff from Egypt, where they indicated a majority that Arabicization can only be applied when reliable translated medical books are made available. They also expressed concerns about Arabicization leading to isolation and hindrance of students’ scientific progress, thus negatively impacting their medical skills (Sabbour et al., 2012). The current study showed that professors from Jordan pointed similar barriers to their students and echoed those from nearby Egypt. In a recent study from Saudi Arabia, the lack of medical resources was the main obstacle to the use of Arabic. Decision-makers also expressed support for a future Arabic curriculum once obstacles are overcome (Alshareef et al., 2018). The most frequently pointed out barriers by medicine university professors in Jordan were English being the official medicine teaching and assessment language, unavailability of medical references that use Arabic terms, and concern that medicine students may not be familiar with Arabic medical terms. Finally, the current study showed a very low rating to some suggested barriers, such as medicine professors feeling less appreciated (23.9%) or ashamed (27.0%) when using Arabic medical terms, which points to the high self-confidence and Arabic language pride among the professors in Jordan.

### 4.4 Impact of gender on Arabicization

It has been previously reported that gender is a significant factor in the acceptability of Arabicized medical terms. It was shown that female practitioners generally had better acceptability than their male counterparts (Halloush, 2000). Similar results were reported in the current study among medicine university professors, where female professors had higher awareness and attitude scores than male colleagues. As for medicine students, male students showed higher awareness and attitude toward Arabicized medical terms. In a study among medicine students from Saudi Arabia, males expressed significantly more preference toward getting their medical education in Arabic because reading in Arabic saved about half of their study time (Al Bar and Assuhaimi, 1996). In alignment, male business students from Jordan showed higher knowledge yet lower attitudes toward Arabicized medical terms than females (Al-abed Al-Haq and Al-Essa, 2016). Therefore, gender seems to be an essential determinant of knowledge and attitudes toward Arabicized terms in general.

### 4.5 Impact of the level of student study on Arabicization

In the current study, the level of students’ study was reported to significantly impact Arabicization. In general, the order of having significantly higher awareness, more positive attitudes, and lower barriers towards Arabicized medical terms was a higher specialty in medicine students, then medicine students in clinical years, and finally, those in pre-clinical years. In agreement, a study from Egypt reported that half of the medicine staff were aware that their medicine students had difficulty with English as a teaching medium, and this was primarily correct for the first year of study (Sabbour et al., 2012). In a study from Saudi Arabia, 74.4% of first-year and 59.9% of third-year medicine students indicated a preference for Arabic as the teaching language (Al Bar and Assuhaimi, 1996).

### 4.6 Impact of the level of income on Arabicization

Interestingly, income was associated with Arabicization, where lower barriers were associated with high income, followed by middle and then low income. This could be related to feeling more empowered financially, which could reflect more courage to carry on with behaviors that are perceived as different or controversial (Jahan et al., 2015; Tong et al., 2021). As well, living in the suburbs was associated with a significantly more favorable attitude toward Arabicized medical terms, which could point to being more attached to the traditional social values of the Arabic culture of Jordan and being less exposed to foreignization when living in the more harmonized and probably, monotonous suburban environment.

### 4.7 Impact of language proficiency on Arabicization

To our knowledge, the current study is the first to correlate language proficiency with awareness, attitudes, and barriers toward Arabicization. Having another language besides Arabic as a mother tongue resulted in significantly more awareness and a more favorable attitude, yet more barriers than those with only Arabic as a mother tongue. In agreement, mixed English and Arabic school study mediums resulted in significantly better awareness, less favorable attitude, and more barriers than pure Arabic or English school study mediums. This is expected in light of previous reports indicating that individuals with more than one mother language have more difficulty grasping artworks and poetry of their mother languages. In other words, exposure to more than one language with its inherently impeded cultural aspects could pose more barriers toward activities of purely single-language centered education or skills (Yazici et al., 2010). Finally, and as expected, medicine students with more Arabic proficiency were associated with significantly higher awareness and more favorable attitudes toward Arabicization. The vice versa applies to English language proficiency.

This study posed five questions regarding changes in awareness, attitude, and barriers toward Arabicized medical terms among medicine students and their professors. Through the quantitative analysis of the research tools, the study managed to answer these questions. First, as per the results of the current study, there seems to be a positive shift in the attitudes of the medical community toward the Arabicization of medical terms. In that regard, the current results showed noticeably improved attitudes among medicine students. The present study also showed generally positive attitudes toward Arabicization among medicine university professors. Second, changes were observed in the acceptability of Arabicized medical terms. Both medicine students and their professors showed over 50% positive agreement with their knowledge, evaluation, usage, and proficiency of Arabicized medical terms. Yet, they showed significantly lower adoption of Arabicized medical terms. Additionally, University professors reported significantly lower rates of Arabicized medical terms proficiency compared to knowledge, evaluation, and usage criteria. Third, the current study showed overall positive acceptability of Arabicized medical terms among medicine students and their professors. Fourth, among medicine students, factors influencing preferences toward Arabicization of medical terms were gender, income, place of living, level of medicine study, having more than one mother tongue, and language proficiency. As for university professors, factors that influenced preferences toward Arabicization of medical terms were gender and English language proficiency. Finally, as per the authors’ site visit and inspection of the webpage of the Jordan Arabic language forum, no change was observed in its procedures and policies as compared to those described in previous studies. Results from the currently studied sample showed the unavailability of valuable medical references that use Arabicized medical terms as one of the most prominent barriers toward Arabicization among medicine students, which was in concordance with their professors. This barrier indicates the need for more efforts from the Arabic language forum to coordinate and push forward the endeavors to avail such references using Arabicized medical terms.

## 5 Conclusion

This study showed a general improvement in awareness/acceptability and more positive attitudes among Medicine students toward Arabicized medical terms. The most common perceived barriers toward Arabicization among medicine students were that teaching and assessments (exams, quizzes, projects, etc.) in English and the unavailability of valuable medical references that use Arabic terms. In addition, several demographic variables were associated with acceptability, attitudes, and/or barriers toward Arabicized terms among medicine students, including gender, income, place of living, level of medicine study, having more than one mother tongue, and language proficiency. On the other hand, medicine university professors showed acceptable awareness and generally positive attitudes toward Arabicized terms. Still, the most frequently cited barriers among medicine professors were in concordance with those pointed out by medicine students, which indicates the validity of these barriers. Finally, gender and English language proficiency were the only factors associated with acceptability, attitudes, and/or barriers toward Arabicized terms among medicine university professors.

This study represents another step toward assessing and evaluating the process of Arabicization in higher education in general and medical sciences in particular. In addition, it provides crucial information to medical and higher education policymakers about the likelihood of success in implementing medical education for students in their native tongue to boost their academic performance and delineate any language barrier they face. In line with the findings of this study, we suggest implementing targeted interventional plans to raise awareness, create more positive attitudes, and delineate barriers. These plans can be directed toward subgroups of the medical community based on the results presented in the current study.

In another respect, the current study has a few limitations. One is that the present study depended on self-reported questionnaires, where self-idealism is an inherited limitation that cannot be avoided. Another inherited limitation is the use of closed-ended questions, which provided the advantage of recruiting a large number of study participants with the ability to get highly consistent and easily quantifiable data. Yet, during the actual data collection of the current study, we could not provide study participants with the freedom to express their thoughts. To remedy that, the authors carried out focus groups discussion during the validation stage of the study questionnaires, where the pilot study participants were encouraged to express their thoughts. Those were incorporated into the final version of the study questionnaires, thus, ensuring the most comprehensive choices for each of the items of the study questionnaires. Moreover, we view the scope of the current study to include only medical terms and to be solely dedicated to medicine students and their professors. Examining the Arabicization of other scientific terms could be a future direction of the current study. In the present study, evaluation of awareness, attitudes, acceptability, and possible barriers toward using Arabicized medical terms was meant to be done in those with Arabic as their native tongue and who lived most of their life in Arabic speaking medium. This is because the after-mentioned criteria represent most medicine students’ bodies and faculty members in Jordan. Those who have lived in an English-speaking country for more than 16 years would have superior English language proficiency. Thus, they are expected to have a disturbing view, especially regarding using Arabicized medical terms for learning or teaching. Such groups could be studied in a separate future study as they are viewed as a district population with different characteristics.

## Declarations

### Author contribution statement

Dalal Al-Zubi, MA: Performed the experiments; Contributed reagents, materials, analysis tools or data; Wrote the paper.

Ahmad El-Sharif, PhD: Conceived and designed the experiments; Wrote the paper.

Karem Alzoubi, PhD: Analyzed and interpreted the data; Contributed reagents, materials, analysis tools or data; Wrote the paper.

### Funding statement

This research did not receive any specific grant from funding agencies in the public, commercial, or not-for-profit sectors.

### Data availability statement

Data will be made available on request.

### Declaration of interest's statement

The authors declare no conflict of interest.

### Additional information

No additional information is available for this paper.

## Appendix A Supplementary data

Appendcies.docx Appendcies.docx
