# Supplementary material for: Changes in the attitudes of professors and students of medicine towards arabicizing medical terms in the faculties of medicine: A study from Jordan
Source: Heliyon. 2022 Dec 6;8(12):e12022. doi: 10.1016/j.heliyon.2022.e12022 (PMC9747590; doi:10.1016/j.heliyon.2022.e12022)
Supplement: Appendcies.docx [file mmc1.docx]

**Appendix I: Students Questionnaire**

**Attitudes of Professors and Students of Medicine towards Arabicizing Medical Terms in the Faculties of Medicine in Jordan**

Consent Form to participate in scientific research

Dear student.......

You are invited to participate in a scientific study entitled “Attitudes of Professors and Students of Medicine towards Arabicizing Medical Terms in the Faculties of Medicine in Jordan”. This study is being carried out by a master's student, Dalal Al-Zubi, from Al al-Bayt University.

Knowing that there are no risks that you may be exposed to, or any material cost as a result of participating in this study. The information that you will provide us with will be used for scientific research purposes by researchers only, and this information will be treated with complete confidentiality.

Filling out the questionnaire takes 8-12 minutes from you, and there is no need to write your name on it.

Your participation in this study is voluntary. When you fill out these forms and receive the questionnaire, you agree to participate in this study voluntarily. You can refrain from answering any question that you do not wish to answer. If you have questions, you can contact the student’s supervisor from the English Department at Al al-Bayt University.

After reviewing the study procedures and answering my inquiries from the researchers, I agree to participate in this study.

Subscriber name: Date: Subscriber signature:

Witness's signature: Date:

| **Part One: General information about the participant** |
| --- |

First of all, we would like to thank you for agreeing to fill out the questionnaire

Your participation in filling out the questionnaire is voluntary, and you can opt out if you do not wish to participate. You only need to notify the administrator when distributing the questionnaire.

This questionnaire includes four parts. The first part asks general questions

1- In which university are you studying?

Ο Jordan University of Science and Technology

Ο The University of Jordan

Ο The Hashemite University

Ο Yarmouk University

Ο Al-Balqa' Applied University

Ο Mutah University

2- If you are a student at the undergraduate level (bachelor), what level are you at?

Ο First year

Ο Second year

Ο Third year

Ο Fourth year

Ο Higher than the fourth and less than the seventh

Ο High specialization

3- How old are you?

The answer: ..................................

4- Your gender is

Ο male

Ο female

5- What is the highest educational attainment that your father or guardian (male) holds?

Ο Less than tenth grade

Ο Tenth grade

Ο High School

Ο Undergraduate level

Ο Master's degree

Ο PhD degree

What is the approximate monthly household income of your family?

Ο Less than 300 Jordanian dinars

Ο 300-500 JD

Ο 501-700 JOD

Ο 701-900 JOD

Ο 901-1100 Jordanian dinars

Ο Above 1100 JD

7- Where does your family currently live?

Ο City

Ο Village

Ο Badia

8- What is your native language?

Ο Arabic

Ο English

Ο Other: …………………………………

(You can choose more than one answer)

6- The language of study in the school (before university):

Ο Arabic

Ο English

Ο Other: …………………………………

(You can choose more than one answer)

8- Your skill level in the following languages:

|  | Excellent | Very Good | Good | Fair | Weak |
| --- | --- | --- | --- | --- | --- |
| Arabic | Ο | Ο | Ο | Ο | Ο |
| English | Ο | Ο | Ο | Ο | Ο |

| **Part II: Awareness, knowledge (acceptability) of Arabicized medical terms** |
| --- |

In this section, five groups of Arabized medical terms will be presented, and under each group there are questions that show your knowledge of these terms. To please answer the questions after each group:

For the below sample of Arabized medical terms and their English equivalents, please answer the following questions:

|  | **Strongly agree** | **Agree** | **Neutral** | **Disagree** | **Strongly disagree** |
| --- | --- | --- | --- | --- | --- |
| I know what most of the medical terms expressed above mean | Ο | Ο | Ο | Ο | Ο |
| I have previously used most of the above expressed terms | Ο | Ο | Ο | Ο | Ο |
| Most of the Arabized terms above show the original meaning (in a way that parallels the original term). | Ο | Ο | Ο | Ο | Ο |
| I think that I am able to use most of the above expressed terms within the correct context and accurately | Ο | Ο | Ο | Ο | Ο |
| I use most of the above expressions frequently and frequently | Ο | Ο | Ο | Ο | Ο |

For the below sample of Arabized medical terms and their English equivalents, please answer the following questions:

|  | **Strongly agree** | **Agree** | **Neutral** | **Disagree** | **Strongly disagree** |
| --- | --- | --- | --- | --- | --- |
| I know what most of the medical terms expressed above mean | Ο | Ο | Ο | Ο | Ο |
| I have previously used most of the above expressed terms | Ο | Ο | Ο | Ο | Ο |
| Most of the Arabized terms above show the original meaning (in a way that parallels the original term). | Ο | Ο | Ο | Ο | Ο |
| I think that I am able to use most of the above expressed terms within the correct context and accurately | Ο | Ο | Ο | Ο | Ο |
| I use most of the above expressions frequently and frequently | Ο | Ο | Ο | Ο | Ο |

For the below sample of Arabized medical terms and their English equivalents, please answer the following questions:

| **The Arabicized Medical term** | **The English term** |
| --- | --- |
| التهاب الاسناخ | Alveolitis |
| الاعتلال الذاتي | Idiopathic |
| ورمي | Neoplastic |
| ألم عضلي | Myalgia |
| ورم ميلانيني | Melanoma |
| صفراء | Bile |
| تحصي صفراوي | Cholelithiasis |
| أم الدم | Aneurysm |
| خُثار | Thrombosis |
| الضهى/انقطاع الحيض | Amenorrhea |
| التهاب الاسناخ | Alveolitis |

|  | **Strongly agree** | **Agree** | **Neutral** | **Disagree** | **Strongly disagree** |
| --- | --- | --- | --- | --- | --- |
| I know what most of the medical terms expressed above mean | Ο | Ο | Ο | Ο | Ο |
| I have previously used most of the above expressed terms | Ο | Ο | Ο | Ο | Ο |
| Most of the Arabized terms above show the original meaning (in a way that parallels the original term). | Ο | Ο | Ο | Ο | Ο |
| I think that I am able to use most of the above expressed terms within the correct context and accurately | Ο | Ο | Ο | Ο | Ο |
| I use most of the above expressions frequently and frequently | Ο | Ο | Ο | Ο | Ο |

For the below sample of Arabized medical terms and their English equivalents, please answer the following questions:

|  | **Strongly agree** | **Agree** | **Neutral** | **Disagree** | **Strongly disagree** |
| --- | --- | --- | --- | --- | --- |
| I know what most of the medical terms expressed above mean | Ο | Ο | Ο | Ο | Ο |
| I have previously used most of the above expressed terms | Ο | Ο | Ο | Ο | Ο |
| Most of the Arabized terms above show the original meaning (in a way that parallels the original term). | Ο | Ο | Ο | Ο | Ο |
| I think that I am able to use most of the above expressed terms within the correct context and accurately | Ο | Ο | Ο | Ο | Ο |
| I use most of the above expressions frequently and frequently | Ο | Ο | Ο | Ο | Ο |

For the below sample of Arabized medical terms and their English equivalents, please answer the following questions:

| **The Arabicized Medical term** | **The English term** |
| --- | --- |
| استئصال الزائدة/اللاحقة | Appendectomy |
| فغر الرغامى | Tracheostomy |
| تنظير الحلق | Laryngoscopy |
| فغر المعدة | Gastrostomy |
| استئصال الثدي | Mastectomy |
| استئصال الطحال | Splenectomy |
| استئصال اللوزتين | Tonsillectomy |
| رأب الأنف | Rhinoplasty |
| رأب الطبلة | Tympanoplasty |
| رأب الرحم | Uteroplasty |

|  | **Strongly agree** | **Agree** | **Neutral** | **Disagree** | **Strongly disagree** |
| --- | --- | --- | --- | --- | --- |
| I know what most of the medical terms expressed above mean | Ο | Ο | Ο | Ο | Ο |
| I have previously used most of the above expressed terms | Ο | Ο | Ο | Ο | Ο |
| Most of the Arabized terms above show the original meaning (in a way that parallels the original term). | Ο | Ο | Ο | Ο | Ο |
| I think that I am able to use most of the above expressed terms within the correct context and accurately | Ο | Ο | Ο | Ο | Ο |
| I use most of the above expressions frequently and frequently | Ο | Ο | Ο | Ο | Ο |

| **Part III: Attitudes toward Arabicized Medical terms** |
| --- |

You will be asked a set of questions about your attitudes and attitudes towards the Arabization of medical terms. Please choose the answer that expresses your agreement with the question posed.

My attitude towards the use of Arabized medical terms is as follows:

|  | **Strongly agree** | **Agree** | **Neutral** | **Disagree** | **Strongly disagree** |
| --- | --- | --- | --- | --- | --- |
| My self-confidence increases when using Arabicized medical terms. | Ο | Ο | Ο | Ο | Ο |
| My belonging to Arabic language is the motive to accept Arabicized medical terms. | Ο | Ο | Ο | Ο | Ο |
| My Islamic religion is the motive to accept Arabicized medical terms. | Ο | Ο | Ο | Ο | Ο |
| I think that The Arabicized medical terms are better than the English medical terms regarding the transformation of ideas and information. | Ο | Ο | Ο | Ο | Ο |
| I think that arabicized terms facilitates communication with my colleagues | Ο | Ο | Ο | Ο | Ο |
| I think that unifying the Arabicized medical terms helps in spreading them in the Arab World. | Ο | Ο | Ο | Ο | Ο |
| I think that the shorter the syllables are for the Arabicized medical terms the more they become distributed. | Ο | Ο | Ο | Ο | Ο |
| I think that the Arabicized medical terms are clear and precise. | Ο | Ο | Ο | Ο | Ο |
| I think that some of the Arabicized medical terms need to be developed and rephrased. | Ο | Ο | Ο | Ο | Ο |
| My colleagues respect me when using Arabicized medical terms. | Ο | Ο | Ο | Ο | Ο |
| I think that the Arabicized medical terms help the Arabic language to cope with the developments in the contemporary life. | Ο | Ο | Ο | Ο | Ο |
| In my discussions with my colleagues, I frequently use the Arabicized medical terms. | Ο | Ο | Ο | Ο | Ο |
| I am seeking to spread and develop the Arabicized medical terms. | Ο | Ο | Ο | Ο | Ο |
|  |  |  |  |  |  |

| **Part III: Barriers toward Arabicized Medical terms** |
| --- |

You will be asked a set of questions about your perception of the barriers and obstacles that contribute to limiting the spread of Arabized medical terms, please kindly choose the answer that expresses your agreement with the question posed.

Expected barriers to the use of Arabized medical terms include:

|  | **Strongly agree** | **Agree** | **Neutral** | **Disagree** | **Strongly disagree** |
| --- | --- | --- | --- | --- | --- |
| I am not familiar with Arabic medical terms | Ο | Ο | Ο | Ο | Ο |
| I am afraid that my colleagues are not familiar with Arabic medical terms | Ο | Ο | Ο | Ο | Ο |
| I would feel less appreciated when I use Arabic medical terms | Ο | Ο | Ο | Ο | Ο |
| Arabic medical terms are lagging in terms of providing the precise meaning | Ο | Ο | Ο | Ο | Ο |
| Arabic medical terms are confusing to me | Ο | Ο | Ο | Ο | Ο |
| I am not confident that I will be able to use Arabic medical terms in the correct context | Ο | Ο | Ο | Ο | Ο |
| I am afraid that the use of Arabic medical terms will not be acceptable by my colleagues | Ο | Ο | Ο | Ο | Ο |
| I would feel ashamed to be the only one using Arabic medical terms | Ο | Ο | Ο | Ο | Ο |
| English Language is the language of education | Ο | Ο | Ο | Ο | Ο |
| Being used to Arabic medical terms could compromise my study progress and scores in later years as all my future study is expected to be using English medical terms | Ο | Ο | Ο | Ο | Ο |
| All my assignments, exams and project require the use of English medical terms | Ο | Ο | Ο | Ο | Ο |
| There is no valuable medical references that use Arabic terms | Ο | Ο | Ο | Ο | Ο |
| The references required by my university courses are all in English and use English medical terms | Ο | Ο | Ο | Ο | Ο |
| I afraid that I will not score well in international medical proficiency and qualifying exams | Ο | Ο | Ο | Ο | Ο |
| The continued use of Arabic medical terms could compromise my future study plans abroad | Ο | Ο | Ο | Ο | Ο |
|  |  |  |  |  |  |

**Appendix II: Academics Questionnaire**

**Attitudes of Professors and Students of Medicine towards Arabicizing Medical Terms in the Faculties of Medicine in Jordan**

Consent Form to participate in scientific research

Dear Colleague.......

You are invited to participate in a scientific study entitled “Attitudes of Professors and Students of Medicine towards Arabicizing Medical Terms in the Faculties of Medicine in Jordan”. This study is being carried out by a master's student, Dalal Al-Zubi, from Al al-Bayt University.

Knowing that there are no risks that you may be exposed to, or any material cost as a result of participating in this study. The information that you will provide us with will be used for scientific research purposes by researchers only, and this information will be treated with complete confidentiality.

Filling out the questionnaire takes 8-12 minutes from you, and there is no need to write your name on it.

Your participation in this study is voluntary. When you fill out these forms and receive the questionnaire, you agree to participate in this study voluntarily. You can refrain from answering any question that you do not wish to answer. If you have questions, you can contact the student’s supervisor from the English Department at Al al-Bayt University.

After reviewing the study procedures and answering my inquiries from the researchers, I agree to participate in this study.

Subscriber name: Date: Subscriber signature:

Witness's signature: Date:

| **Part One: General information about the participant** |
| --- |

First of all, we would like to thank you for agreeing to fill out the questionnaire

Your participation in filling out the questionnaire is voluntary, and you can opt out if you do not wish to participate. You only need to notify the administrator when distributing the questionnaire.

This questionnaire includes four parts. The first part asks general questions

1- In which university do you work?

Ο Jordan University of Science and Technology

Ο The University of Jordan

Ο The Hashemite University

Ο Yarmouk University

Ο Al-Balqa' Applied University

Ο Mutah University

2- How old are you?

the answer: ..................................

3- Your gender is

Ο male

Ο female

4- How much experience do you have in practicing medicine and teaching medical specialties?

Ο Less than 5 years old

Ο More than 5 years and less than 15 years

Ο More than 15 years and less than 25 years

Ο More than 25 years and less than 35 years

Ο More than 35 years old

5- What is your mother tongue?

Ο Arabic

Ο English

Ο Other: …………………………………

(You can choose more than one answer)

6- The language of study or training in the highest degree you obtained is:

Ο Arabic

Ο English

Ο Other: …………………………………

7- The country of study or training for the highest scientific degree you obtained is:

Ο English-speaking country as a mother tongue

Ο A country that speaks Arabic as a mother tongue

Ο A country that speaks another language as a mother tongue

8- Your skill level in the following languages:

|  | Excellent | Very Good | Good | Fair | Weak |
| --- | --- | --- | --- | --- | --- |
| Arabic | Ο | Ο | Ο | Ο | Ο |
| English | Ο | Ο | Ο | Ο | Ο |

| **Part II: Awareness, knowledge (acceptability) of Arabicized medical terms** |
| --- |

In this section, five groups of Arabized medical terms will be presented, and under each group there are questions that show your knowledge of these terms. To please answer the questions after each group:

For the below sample of Arabized medical terms and their English equivalents, please answer the following questions:

|  | **Strongly agree** | **Agree** | **Neutral** | **Disagree** | **Strongly disagree** |
| --- | --- | --- | --- | --- | --- |
| I know what most of the medical terms expressed above mean | Ο | Ο | Ο | Ο | Ο |
| I have previously used most of the above expressed terms | Ο | Ο | Ο | Ο | Ο |
| Most of the Arabized terms above show the original meaning (in a way that parallels the original term). | Ο | Ο | Ο | Ο | Ο |
| I think that I am able to use most of the above expressed terms within the correct context and accurately | Ο | Ο | Ο | Ο | Ο |
| I use most of the above expressions frequently and frequently | Ο | Ο | Ο | Ο | Ο |

For the below sample of Arabized medical terms and their English equivalents, please answer the following questions:

|  | **Strongly agree** | **Agree** | **Neutral** | **Disagree** | **Strongly disagree** |
| --- | --- | --- | --- | --- | --- |
| I know what most of the medical terms expressed above mean | Ο | Ο | Ο | Ο | Ο |
| I have previously used most of the above expressed terms | Ο | Ο | Ο | Ο | Ο |
| Most of the Arabized terms above show the original meaning (in a way that parallels the original term). | Ο | Ο | Ο | Ο | Ο |
| I think that I am able to use most of the above expressed terms within the correct context and accurately | Ο | Ο | Ο | Ο | Ο |
| I use most of the above expressions frequently and frequently | Ο | Ο | Ο | Ο | Ο |

For the below sample of Arabized medical terms and their English equivalents, please answer the following questions:

| **The Arabicized Medical term** | **The English term** |
| --- | --- |
| التهاب الاسناخ | Alveolitis |
| الاعتلال الذاتي | Idiopathic |
| ورمي | Neoplastic |
| ألم عضلي | Myalgia |
| ورم ميلانيني | Melanoma |
| صفراء | Bile |
| تحصي صفراوي | Cholelithiasis |
| أم الدم | Aneurysm |
| خُثار | Thrombosis |
| الضهى/انقطاع الحيض | Amenorrhea |
| التهاب الاسناخ | Alveolitis |

|  | **Strongly agree** | **Agree** | **Neutral** | **Disagree** | **Strongly disagree** |
| --- | --- | --- | --- | --- | --- |
| I know what most of the medical terms expressed above mean | Ο | Ο | Ο | Ο | Ο |
| I have previously used most of the above expressed terms | Ο | Ο | Ο | Ο | Ο |
| Most of the Arabized terms above show the original meaning (in a way that parallels the original term). | Ο | Ο | Ο | Ο | Ο |
| I think that I am able to use most of the above expressed terms within the correct context and accurately | Ο | Ο | Ο | Ο | Ο |
| I use most of the above expressions frequently and frequently | Ο | Ο | Ο | Ο | Ο |

For the below sample of Arabized medical terms and their English equivalents, please answer the following questions:

|  | **Strongly agree** | **Agree** | **Neutral** | **Disagree** | **Strongly disagree** |
| --- | --- | --- | --- | --- | --- |
| I know what most of the medical terms expressed above mean | Ο | Ο | Ο | Ο | Ο |
| I have previously used most of the above expressed terms | Ο | Ο | Ο | Ο | Ο |
| Most of the Arabized terms above show the original meaning (in a way that parallels the original term). | Ο | Ο | Ο | Ο | Ο |
| I think that I am able to use most of the above expressed terms within the correct context and accurately | Ο | Ο | Ο | Ο | Ο |
| I use most of the above expressions frequently and frequently | Ο | Ο | Ο | Ο | Ο |

For the below sample of Arabized medical terms and their English equivalents, please answer the following questions:

| **The Arabicized Medical term** | **The English term** |
| --- | --- |
| استئصال الزائدة/اللاحقة | Appendectomy |
| فغر الرغامى | Tracheostomy |
| تنظير الحلق | Laryngoscopy |
| فغر المعدة | Gastrostomy |
| استئصال الثدي | Mastectomy |
| استئصال الطحال | Splenectomy |
| استئصال اللوزتين | Tonsillectomy |
| رأب الأنف | Rhinoplasty |
| رأب الطبلة | Tympanoplasty |
| رأب الرحم | Uteroplasty |

|  | **Strongly agree** | **Agree** | **Neutral** | **Disagree** | **Strongly disagree** |
| --- | --- | --- | --- | --- | --- |
| I know what most of the medical terms expressed above mean | Ο | Ο | Ο | Ο | Ο |
| I have previously used most of the above expressed terms | Ο | Ο | Ο | Ο | Ο |
| Most of the Arabized terms above show the original meaning (in a way that parallels the original term). | Ο | Ο | Ο | Ο | Ο |
| I think that I am able to use most of the above expressed terms within the correct context and accurately | Ο | Ο | Ο | Ο | Ο |
| I use most of the above expressions frequently and frequently | Ο | Ο | Ο | Ο | Ο |

| **Part III: Attitudes toward Arabicized Medical terms** |
| --- |

You will be asked a set of questions about your attitudes and attitudes towards the Arabization of medical terms. Please choose the answer that expresses your agreement with the question posed.

My attitude towards the use of Arabized medical terms is as follows:

|  | **Strongly agree** | **Agree** | **Neutral** | **Disagree** | **Strongly disagree** |
| --- | --- | --- | --- | --- | --- |
| My self-confidence increases when using Arabicized medical terms. | Ο | Ο | Ο | Ο | Ο |
| My belonging to Arabic language is the motive to accept Arabicized medical terms. | Ο | Ο | Ο | Ο | Ο |
| My Islamic religion is the motive to accept Arabicized medical terms. | Ο | Ο | Ο | Ο | Ο |
| I think that The Arabicized medical terms are better than the English medical terms regarding the transformation of ideas and information. | Ο | Ο | Ο | Ο | Ο |
| I think that arabicized terms facilitates communication with my students | Ο | Ο | Ο | Ο | Ο |
| I think that unifying the Arabicized medical terms helps in spreading them in the Arab World. | Ο | Ο | Ο | Ο | Ο |
| I think that the shorter the syllables are for the Arabicized medical terms the more they become distributed. | Ο | Ο | Ο | Ο | Ο |
| I think that the Arabicized medical terms are clear and precise. | Ο | Ο | Ο | Ο | Ο |
| I think that some of the Arabicized medical terms need to be developed and rephrased. | Ο | Ο | Ο | Ο | Ο |
| My colleagues respect me when using Arabicized medical terms. | Ο | Ο | Ο | Ο | Ο |
| I think that the Arabicized medical terms help the Arabic language to cope with the developments in the contemporary life. | Ο | Ο | Ο | Ο | Ο |
| In my discussions with my colleagues, I frequently use the Arabicized medical terms. | Ο | Ο | Ο | Ο | Ο |
| I am seeking to spread and develop the Arabicized medical terms. | Ο | Ο | Ο | Ο | Ο |
|  |  |  |  |  |  |

| **Part III: Barriers toward Arabicized Medical terms** |
| --- |

You will be asked a set of questions about your perception of the barriers and obstacles that contribute to limiting the spread of Arabized medical terms, please kindly choose the answer that expresses your agreement with the question posed.

Expected barriers to the use of Arabized medical terms include:

|  | **Strongly agree** | **Agree** | **Neutral** | **Disagree** | **Strongly disagree** |
| --- | --- | --- | --- | --- | --- |
| I am not familiar with Arabic medical terms | Ο | Ο | Ο | Ο | Ο |
| I am afraid that my colleagues and coworkers are not familiar with Arabic medical terms | Ο | Ο | Ο | Ο | Ο |
| I am afraid that my students are not familiar with Arabic medical terms | Ο | Ο | Ο | Ο | Ο |
| I would feel less appreciated when I use Arabic medical terms | Ο | Ο | Ο | Ο | Ο |
| Arabic medical terms are lagging in terms of providing the precise meaning | Ο | Ο | Ο | Ο | Ο |
| Arabic medical terms are confusing to me | Ο | Ο | Ο | Ο | Ο |
| I am not confident that I will be able to use Arabic medical terms in the correct context | Ο | Ο | Ο | Ο | Ο |
| I am afraid that the use of Arabic medical terms will not be acceptable by my college | Ο | Ο | Ο | Ο | Ο |
| I would feel ashamed to be the only one using Arabic medical terms | Ο | Ο | Ο | Ο | Ο |
| English Language is the language of education | Ο | Ο | Ο | Ο | Ο |
| Being used to Arabic medical terms could compromise my study progress and scores in later years as all my future study is expected to be using English medical terms | Ο | Ο | Ο | Ο | Ο |
| All my assignments, exams and project require the use of English medical terms | Ο | Ο | Ο | Ο | Ο |
| There is no valuable medical references that use Arabic terms | Ο | Ο | Ο | Ο | Ο |
| The references required by my university courses are all in English and use English medical terms | Ο | Ο | Ο | Ο | Ο |
|  |  |  |  |  |  |
